# Evaluation of the Peri-Implant Bone Level around Platform-Switched Dental Implants: A Retrospective 3-Year Radiographic Study

**DOI:** 10.3390/ijerph16142570

**Published:** 2019-07-18

**Authors:** Yu Hwa Pan, His Kuei Lin, Jerry C-Y Lin, Yung-Szu Hsu, Yi-Fan Wu, Eisner Salamanca, Wei-Jen Chang

**Affiliations:** 1 School of Dentistry, College of Oral Medicine, Taipei Medical University, Taipei 10692, Taiwan; 2 Department of Dentistry, Chang Gung Memorial Hospital, Taipei 10488, Taiwan; 3 Graduate Institute of Dental & Craniofacial Science, Chang Gung University, Taoyuan 33343, Taiwan; 4 School of Dentistry, College of Medicine, China Medical University, Taichung 40150, Taiwan; 5 Dental Department, Taipei Medical University, Shuang-Ho Hospital, New Taipei City 23557, Taiwan; 6 Department of Oral Medicine, Infection and Immunity, Harvard School of Dental Medicine, Boston, MA 02115, USA

**Keywords:** peri-implant bone level, platform switching, dental implant

## Abstract

Objective: To describe remodeling of the mesial and distal marginal bone level around platform-switched (PS) and platform-matched (PM) dental implants that were sandblasted with large grit and etched with acid over a three-year period. Materials and Methods: Digital periapical radiographs were obtained at the following time-points: during Stage I of the surgical placement of dental implants, before loading, immediately after loading (baseline), and one, three, six, 12, and 36 months after loading for measuring the horizontal and vertical marginal bone levels. Results: Sixty implants were successfully osseointegrated during the overall observation period. Vertical marginal bone levels for the PS and PM dental implants were 0.78 ± 0.77 and 0.98 ± 0.81 mm, respectively, whereas the horizontal marginal bone levels for the PS and PM implants were 0.84 ± 0.45 and 0.98 ± 0.68 mm, respectively. During the time leading up to the procedure until 36 months after the procedure, the average vertical marginal bone level resulted in less bone loss for the PS and PM groups—0.96 ± 1.28 and 0.30 ± 1.15 mm, respectively (*p* < 0.05). The mean levels of the horizontal marginal bone also showed increases of 0.48 ± 1.01 mm in the PS and 0.37 ± 0.77 mm in the PM groups from the time before loading until 36 months after the procedure. However, these increases were not statistically significant (*p* > 0.05). Conclusion: PS dental implants appeared to be more effective than PM implants for minimizing the mean marginal vertical and horizontal marginal bone loss during the three-year period. Regardless of which abutment connection was used, the dental implant in the present retrospective investigation exhibited minimal marginal bone remodeling, thus indicating long-term stability.

## 1. Introduction

Implant therapy is a reliable and predictable tool for dental rehabilitation that requires multiple factors for the maintenance of long-term treatment success and aesthetics. Gardner and Lazzara introduced the concept of platform switching (PS), which refers to the situation where a larger-diameter implant is combined with a narrower abutment, resulting in a shift of the implant abutment gap away from the implant shoulder [1,2,3,4]. The benefits and feasibility of PS have been discussed in several studies, with a report stating that PS implants decreased crestal bone loss compared with platform-matched (PM) implants [1,3,5]. One potential factor behind the ability of PS implants to minimize changes in crestal bone height includes the load distribution of the implant and the concentration of forces. This causes the implant to sink sub-crestally during its placement and reduces localized inflammation within the soft tissue [3].

Platform-switching is considered to be a meaningful factor in limiting the resorption of crestal bone and ensuring the success of dental implants in the long term [6]. The biomechanical hypotheses supporting platform switching for crestal bone preservation indicate that the stress level in the cervical bone area is reduced by directing the forces of occlusal loading from the interface between the crestal bone and the implant to the axis of the implant. However, this method has the disadvantage of increasing stress within the abutment or abutment screw [7]. Another hypothesis is that the cause of bone loss during this procedure indicates that it may be caused by the biologic width reestablishment found in implants placed below the crestal bone [8]. Additionally, when inflammatory cells infiltrate the implant–abutment connection, the volume of crestal bone is reduced in the peri-implant region [9,10,11]. As reported in longitudinal studies, the position of the implant abutment interface relative to the alveolar crest as well as the presence of microgaps between implant components may induce substantial bone loss prior to definitive restoration delivery [12,13,14]. Despite the proposed hypotheses for explaining bone loss, PS has a positive effect on the reduction of bone loss around dental implants, though it comes with indefinite results [1,15].

The evaluation of treatment outcomes has changed in recent years from determining the success of the procedure and/or the survival rates of patients to measuring the long-term stability of the peri-implant tissue using radiographic and clinical parameters [16]. Periapical radiographs are an efficient tool for evaluating mean marginal bone loss, and they are frequently used in routine clinical practice to prevent treatment failure and ensure a favorable long-term prognosis for dental implants. This method of evaluation has been debated, and some studies have shown bone loss in the areas enclosing dental implants. For example, Adell [17] reported the crestal bone loss during the first year after abutment connection was 1.2 mm according to radiographic results. Furthermore, this study showed that the mean vertical bone loss was <0.2 mm annually following function in both the mandible and the maxilla [18]. Subsequently, Albrektsson proposed that a dental implant could be considered successful if there was <1.5 mm of peri-implant crestal bone loss during the first year of placement and if annual bone loss after the first year remained less than <0.2 mm per year [19]. Moreover, several other studies have found that the long-term success of endosseous implants primarily depends on the preservation of surrounding bone support [20,21,22,23,24]. Therefore, radiographic follow-up imaging is essential for the successful maintenance of endosseous implants and for the minimization of the changes in the surrounding bone level. Therefore, the aim of the present study was to radiographically describe changes at the level of the mesial and distal marginal bone surrounding PS and PM dental implants during the three-year period after the restoration.

## 2. Materials and Methods

The study protocol was registered and approved by Taipei Medical University Joint Institutional Review Board (Approval No. N201708047). This retrospective radiographic study was performed following a complete review of the dental records at Taipei Medical University Shuang-Ho Hospital New Taipei, Taiwan. All patient-related information such as gender, age at the time of surgery, reasons for loss, or extraction of natural teeth were collected for this study. In addition, the following information was also recorded: Stage I surgical placement of dental implant before loading (healing abutment placement), immediately after loading (baseline), and 1, 3, 6, 12, and 36 months after loading. In addition, the delivery date for the prosthesis were recorded.

### 2.1. Inclusion and Exclusion Criteria 

All patients older than 18 years old who received a single dental implant were enrolled in the study and considered for undergoing periapical radiograph measurements if they fulfilled the criteria established by the research, science and therapy committee of the American Academy of Periodontology [25]. In addition to the previously established criteria, patients were included if they required a single-tooth extraction and had all-natural dentition on the opposite side of the arch. Other criteria for inclusion in this study included having good systemic health, being a nonsmoker or smoking ≤10 cigarettes/day, having good oral hygiene, having no active infection around the surgical site, having adequate bone tissue to ensure primary implant stability, having the presence of keratinized tissue (KT) ≥ 2 mm at the time of implant placement, having stable posterior occlusion, and having the absence of bruxism/clenching.

Patients were excluded if they had radiation therapy in the head less than 12 months prior to the study, past or present treatment with intravenous amino-bisphosphonates, smoked >10 cigarettes/day, chewed betel nut or tobacco, had alcoholism, were pregnant or breast feeding, were on long-term oral medication, had oral parafunction, had non-treated periodontal disease, were in need of major bone augmentation procedures with autogenous bone or bone substitutes prior to insertion of the implant, had an inability to commit to implant therapy and its maintenance, and/or had the failure or unwillingness to return for follow-up radiography.

### 2.2. Surgical and Prosthetic Procedures

**Figure 1:**
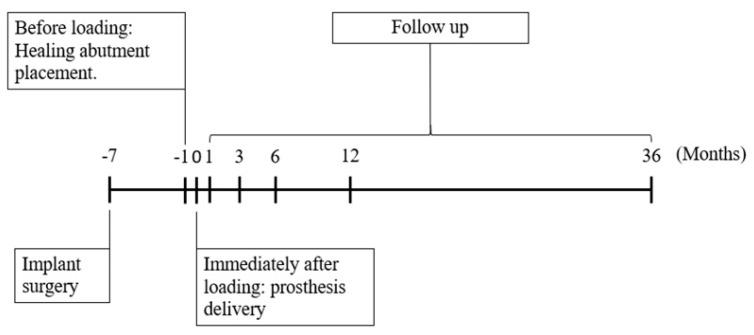
Study timeline.

Several procedures were performed to prevent medical complications when placing the implant and to optimize the availability of soft tissue for primary healing. Six weeks after the teeth were extracted without pathological change in the surrounding bone, dental implants were inserted according to the manufacturer’s surgical protocol. Two types of titanium implants were used in this study. Implantium^®^ (Dentium USA, Cypress, CA, USA) were used for platform switching, and Xive^®^ (DENTSPLY Implants Manufacturing GmbH, Mannheim Germany) for platform matching. Each implant was at least 3.6 mm in width and at least 8 mm in length with internal connections. All implants were inserted until the outer edge of the implant was within 1 mm of the subcrestal bone [3]. Dental implants were left with the healing screw at the time of placement in preparation for a second surgical approach. The presence of keratinized tissue was confirmed during healing and abutment placement. A trained dental surgeon performed the surgical and restorative procedures described in this study. For PS abutments, a horizontal circular mismatch between 0.3 and 0.6 mm was placed between the outer edge of the dental implant and the narrower abutment. Patients underwent 36 months of follow-up examination after delivery, and the placement of permanent individual cemented retained porcelain fused to metal restoration (Figure 1).

### 2.3. Outcome Measurements

The dental implants were considered successful in the absence of pain, infections, neuropathy, paresthesia, and the violation of vital structures. Other factors examined for outcomes included the following: The implant stayed in place, the absence of continuous peri-implant radiolucency, less than 0.2 mm of progressive bone loss after physiologic remodeling the first year of function, and satisfaction with the implant (patient and dentist) [25].

**Figure 2:**
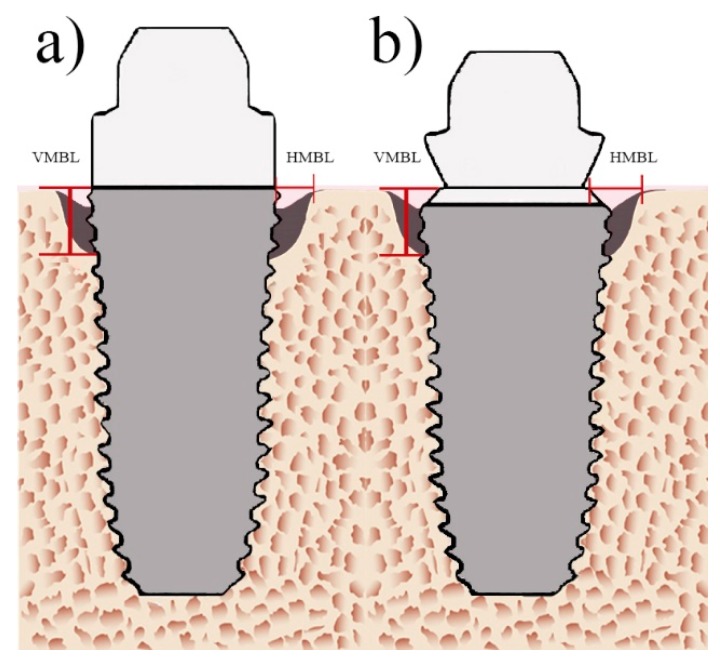
Radiographic measurements of platform-switched implants. (**a**) Platform-matched (PM) dental implants, (**b**) platform-switched (PS) dental implants. Vertical marginal bone loss, VMBL; horizontal marginal bone loss, HMBL.

Patients were recalled for routine evaluation, and digital periapical radiographs were used to image dental implants. Standardized periapical radiographs were performed using a long-cone paralleling technique using commercially available film holders. While radiographs were being taken, it was confirmed that the threaded profile of the implant was visible from the mesial and distal perspectives [19]. Images were taken prior to Stage I of the dental implant surgical placement, before loading (healing abutment placement), immediately after loading (baseline), and 1, 3, 6, 12, and 36 months after loading. The following measurements for each radiographic image were taken of the mesial and distal aspects of the implant:

- Horizontal marginal bone level (HMBL): Horizontal measurement from the implant–abutment interface to the bone crest [26] for PM (Figure 2a) and PS (Figure 2b).
- Vertical marginal bone level (VMBL): Vertical measurement from the implant–abutment interface to the first bone-implant contact [27] for PM (Figure 2a) and PS (Figure 2b).

The ratio of the bone supported surface was calculated using the resorption and apparently osseointegrated areas. The ratio between known dental implant length to vertical marginal bone loss was used to determine the percentage of bone that was in direct contact with the implant. The average mesial and distal measurements were calculated for each dental implant at different time points to determine the extent to which vertical marginal bone loss affected the stability of the implant over the 36-month follow-up period.

EZ-Dental professional imaging software (Asahi Co. Ltd., Tokyo, Japan) was used to calculate all measurements. Radiographs were recorded and collected by two calibrated examiners, and a dental surgeon performed the implant placement. The length calibration tool of the EZ-Dental professional imaging software was used to correct for any deviation in the periapical films. The known diameter and length of the dental implant was used to calibrate the periapical films. Thereafter, the length-measuring tool was used to obtain the mesial and distal measurements of vertical and horizontal bone levels at the different time points.

### 2.4. Statistical Analysis

Microsoft Excel Professional Plus 2016 (Microsoft Software, Redmond, WA, USA) was used for all data analyses. Descriptive statistics and continuous variables are expressed as the mean ± the standard deviation. “Dental implant” was used as the unit for all analyses. Before statistical tests were performed, the Jarque–Bera test was used to test normality. Following normality testing, independent and paired sample *t*-tests were used to analyze data. Comparisons were computed by means with repeated measures within and between groups. *p* < 0.05 was considered statistically significant for all statistical tests.

## 3. Results

**Figure 3:**
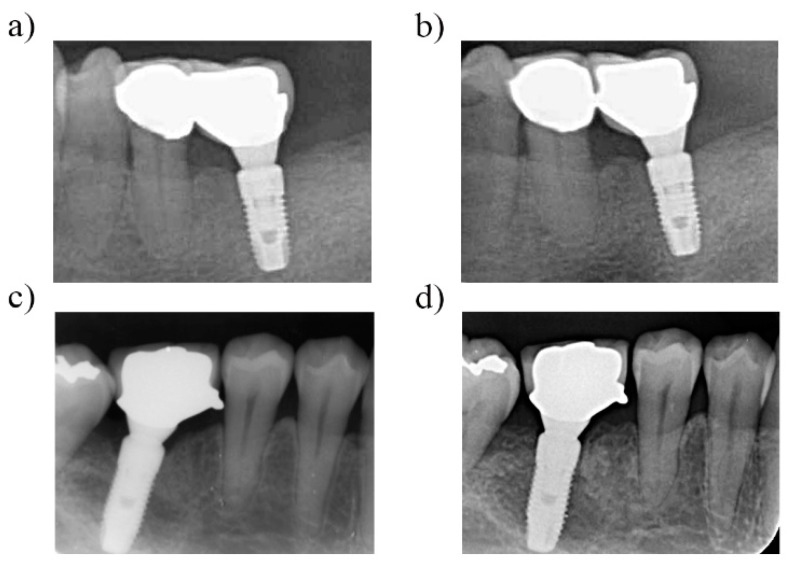
Radiographic follow-up images of dental implants. (**a**) Platform-switched dental implant in lower left area immediately after prosthetic delivery. (**b**) Radiographic view of the same dental implant in the lower left area at a three-year follow-up, with no apparent bone remodeling. (**c**) Platform-switched dental implant in the lower right area immediately after prosthetic delivery, (**d**) Radiographic view of the same dental implant in lower right area at three-year follow-up, with minimal bone remodeling.

In the current study, no implants were lost in either group from the time of implant placement, nor during prosthetic delivery (Figure 3a,c), until after the three-year follow-up (Figure 3b,d). In total, 47 patients between the ages of 28 and 80 years (24 men and 23 women) received 60 dental implants. Of these, 30 PM implants were placed in 24 patients (15 men with a mean age of 53.7 ± 19.7 years and nine women with a mean age of 54.2 ± 26 years), and 30 PS implants were placed in 23 patients (nine men with a mean age of 53.1 ± 28 years and 14 women with a mean age of 54.3 ± 15.7 years) (Table 1). Out of the 30 PM implants placed, 26 (86.67%) were implanted in the posterior region and four (13.33%) were in the anterior region; 25 of these PM implants were in the maxilla, and five (16.67%) were in the mandible. Twenty-eight (93.33%) of the 30 PS implants were placed in the posterior region, only two (6.67%) were placed in the anterior region, 17 (56.67%) were implanted in the mandible, and 13 (43.33%) were placed in the maxilla. The distribution of diameter measurements for all dental implants appears in Table 1. Most PM implants had a diameter of 4.0 mm (46.66%) and 4.5 mm (33.33%), while most PS implants were 4.0 mm (33.33%) and 4.5 mm (43.33%).

**Figure 4:**
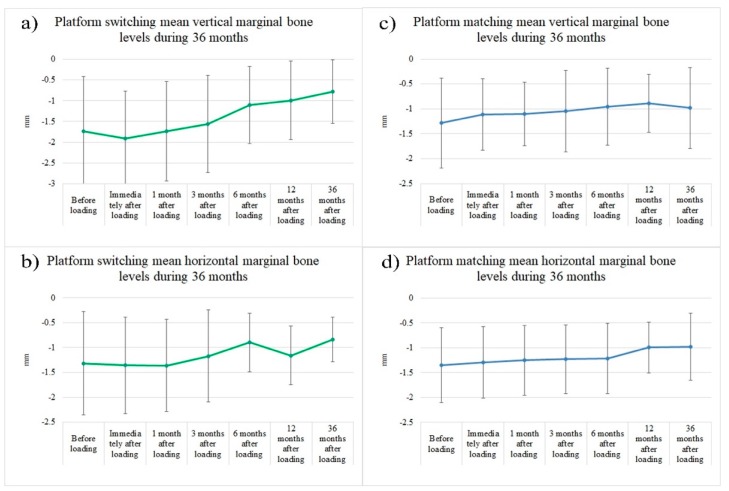
Measurements for mean vertical and horizontal marginal bone loss over the 36-month follow-up period. Variations in the mean vertical and horizontal marginal bone loss before and up to 36 months after loading. (**a**) The mean vertical marginal bone loss measurements over the 36-month period for PS implants. (**b**) The mean horizontal marginal bone loss measurements over the 36-month period for PS implants. (**c**) The mean vertical marginal bone loss measurements over the 36-month period for PM implants. (**d**) The mean horizontal marginal bone loss measurements over the 36-month period for PM implants.

The mean level of the vertical marginal bone before loading the PS implants was 1.74 ± 1.32 mm. Thirty-six months after loading the implant, this level was reduced to 0.78 ± 0.77 mm. A statistically significant difference (*p* < 0.05) was seen among the VMBLs at baseline and at 6, 12, and 36 months after placing the implant (Figure 4, Table 2). The mean HMBL before loading the PS implants was 1.32 ± 1.64 mm, while this level was decreased to 0.84 ± 0.45 mm at 36 months after loading. This change in the horizontal marginal bone level was statistically significant (*p* < 0.05). (Figure 4, Table 2).

The mean variation in the VMBL for PM implants was 1.29 ± 0.90 mm before loading and 0.98 ± 0.81 mm at 36 months after loading. There was only a significant difference (*p* < 0.05) between the baseline and 12 months in regard to the distal and mean measurements (Figure 4c). By contrast, the mean horizontal bone level for PM implants was 1.35 ± 0.75 mm before loading and 0.98 ± 0.68 mm at 36 months after loading. For the horizontal measurements in the PM implants, there were no statistically significant differences between the baseline and the other time points. (Figure 4d).

**Figure 5:**
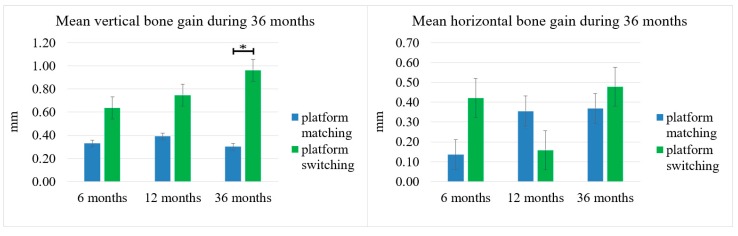
Mean vertical and horizontal bone gain at 6, 12, and 36 months, reported as standard error. Asterisks (*) indicate a statistically significant difference (*p* < 0.05).

The PS implants had the following mean gains in vertical bone: 0.64 ± 1.12 mm at 6 months, 0.75 ± 1.19 mm at 12 months, and 0.96 ± 1.28 mm at 36 months. The mean vertical bone gain from healing abutment placement were as follows in the PM implants: 0.33 ± 1.04 mm at 6 months, 0.39 ± 0.98 mm at 12 months, and 0.30 ± 1.15 mm at 36 months after placement. At 36 months, vertical bone gain was significantly greater in the PS implants compared to the PM implants (Figure 5).

The mean increase in HMBL from baseline to 6, 12, and 36 months in PS implants was 0.42 ± 0.89 mm, 0.16 ± 1.08 mm, and 0.48 ± 1.01 mm, respectively. The mean horizontal bone loss from baseline until 6, 12, and 36 months in the PM implants was 0.14 ± 0.60 mm, 0.35 ± 0.68 mm, 0.37 ± 0.77 mm, respectively, values which were not statistically significant (Figure 5).

**Figure 6:**
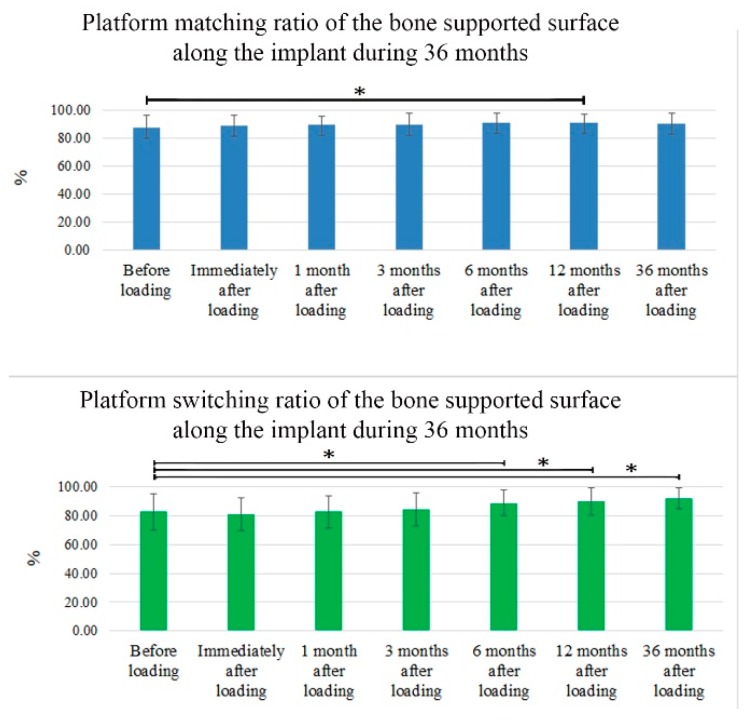
Ratio of the bone supported surface along the implant fixture over the 36-month period. Percentage of bone contact along the implant for 36 months of follow-up in the PM and PS implants. Asterisks (*) indicate a statistically significant difference (*p* < 0.05).

The ratios of the bone supported surface over the 36-month follow-up period are shown in Figure 6. These ratios demonstrate that the PM group averaged 88% ± 8.6% contact before loading but improved to 90% ± 7.54% at 36 months after loading (*p* > 0.05). Meanwhile, contact was 83% ± 12.29% in the PS group before loading, and this ratio improved to 92.40% ± 7.22% at 36 months after loading (*p* < 0.05) (Figure 6).

## 4. Discussion

The aim of the present study was to describe the horizontal and vertical variations in the marginal bone level at the mesial and distal sides around the PS and PM dental implants during the 36-month period following the insertion of the implant.

Implants exhibited osseointegration at three years with a 100% rate of success. The medium-term survival rate in the current study matched the 100% survival rate found in the study by Mendoza-Azpur et al. at one year short term assessment, where mini implants with same surface treatment were used for marginal peri-implant bone level evaluation [28].

These results the justify expectations of successful long-term survival, since there is sufficient evidence that implant losses mainly occur within the first few months after placement [29]. In a five-year retrospective clinical study conducted by Lee et al., 249 of the same type of dental implants used in the present study were evaluated. For this study, the survival rate was 97.1%. The majority of the failed implants were removed between placement and loading [30]. The survival rate in the present study was 100%, which is higher than the 97.1% rate published by Lee et al. The higher survival rate in our study is possibly related to the smaller sample as well as the shorter period of follow-up (three years vs. five years). The study by Lee et al. differed from the present study in that the implants were loaded immediately after Stage I surgery. By contrast, this study followed the original Brånemark concept, where implants were placed during Stage I surgery in a conventional manner and covered. Then, implants were properly restored after osseointegration had occurred. Between the healing period, abutment placement, and the time immediately after loading, all dental implants suffered bone remodeling. However, a statistical analysis showed no significant difference. Furthermore, the PS implants were inserted in a slightly deeper position than the PM implants during the surgical procedure. The bone remodeling and minimal difference in bone level at the time of insertion did not alter the results of the statistical analysis. The PS implants had a higher mean gain in the vertical bone level gain at 6, 12, and 36 months after placement, with a statistically significant increase at three years compared to the PM implants (Figure 5).

Longitudinal studies have reported that peri-implantitis can affect the success rate of dental implants. A 10-year retrospective clinical study using same sandblasted, large grit, and acid-etched dental implants showed that the cumulative survival rate was similar to the present study, with a survival rate of 97.9%. These results demonstrate that these implants have very good long-term stability, and the low percentage of implant failure was due to peri-implantitis, smoking, and bruxism [31]. These failures were not present in our study, possibly due to the exclusion criteria that ruled out patients with these characteristics. It is important to be cautious when treating patients with these characteristics. Furthermore, it is also recommended to perform a more thorough analysis of the dental implants used in this study to better treat peri-implantitis and patients that smoke or have bruxism.

Both the PS and PM implant groups exhibited gains in the mean vertical and HMBLs during the 36-month period of study. The PS implants showed a statistically significant increase in vertical marginal bone gain (0.96 ± 1.28 mm) compared to the PM implants (0.30 ± 1.15 mm) at 36 months. Additionally, the PS implants had a higher ratio of the bone supported surface after six months (*p* < 0.05). Similar results of better-maintained marginal bone levels in PS implants have been reported in previous studies. A meta-analysis by Chrcanovic associated PS implants with significantly lower levels of marginal bone loss in comparison to PM implants. In this study, an increase of the mean difference of the marginal bone level (MBL) between the procedures was observed with an increase in the follow-up time. Furthermore, Chrcanovic et al. advised readers to use caution when interpreting the results of their study due to the presence of uncontrolled confounding factors in the included studies, which also tended to have short follow-up periods [32]. Additionally, positive results for the PS implants compared to the PM implants were attributed to the decreased risk of bacterial microleakage and bone micro-movements associated with the microgap between the abutment–implant interface. This microgap needs to be below the crestal bone so that the PS advantages can be observed. In addition to PS, multiple factors can alter MBLs in a long term, making more controlled studies indispensable [32]. In addition to the medium-term results in our study, the PS concept after 10 years of prosthetic loading enabled satisfactory long-term aesthetic results and avoided less soft tissue shrinkage compared to PM restorations [33,34].

Previous studies have suggested that the position of the peri-implant marginal bone following the delivery of the final prosthetic restoration may be established based on several factors that determine the crestal bone level around dental implants. This requires at least 3 mm of soft tissue, which is necessary for the formation of a biological seal without an increased loss of crestal bone height. In addition to implant surface topography, the position of the implant–abutment junction, where cell infiltration may occur, and its proximity to the crestal bone may induce its loss [3,35]. In the present study, the soft tissue above the dental implants was not measured. Despite the general guideline that dictates a minimum of 3 mm of mucosal thickness for the establishment of a biological width, Berglundh and Lindhe reported greater crestal bone loss when the soft tissue surrounding an implant was intentionally reduced to ≤2 mm [36]. However, other authors had recently described only significant differences in bone loss between PS and PM implant sides at a mean of 4.22 mm with a wide range of 1.50–7.00 mm thicknesses for soft tissue [37]. More studies focusing on the soft tissues surrounding the implant are necessary in the future. This study only included dental implants with ≥2 mm of keratinized tissue at the time of implant placement and only studied changes in bone tissue. A statistically significant difference in the mean gain for vertical bone height between the two groups was observed at 36 months. The mean horizontal bone gain for the PS implants was 0.48 ± 1.01 mm for 36 months, while it was 0.37 ± 0.77 mm for the PM implants (*p* > 0.05). Clinical outcomes such vertical mucosal thickness [35], probing depth, or clinical attachment levels in correlation with confounding factors [32] could explained the range in standard deviations observed in measurements for the mean VMBL. These clinical factors should be taken into consideration for future studies to determine the effect of PS on bone remodeling surrounding the dental implants, rather than only focusing on the results of radiographic imaging.

The dental implants used in this study have been tested in previous studies and demonstrated adequate bone-implant contact and clinical performance as determined by the crestal bone height [38]. Furthermore, the percentage of bone contact along the edge of the implants throughout the 36-month follow-up period (Figure 6) indicated that bone remodeling did not threaten the anchorage or foundation of the implant. Considering the design of the dental implant, the implant–abutment junction was left at the MBL. We found that minimal vertical marginal bone remodeling occurred in all cases, which could be attributed to the remodeling of biological bone that occurs in the first year after the dental implant is loaded. In accordance with the results of a previously published systematic review and meta-analysis, PS for preserving marginal bone around dental implants and a ≥0.4 mm abutment/implant diameter difference were associated with a more favorable bone response [39]. In our study, the abutment/implant diameter difference was 0.6 mm. Despite the medium-term study period of 36 months, minimal crestal bone remodeling with bone gain and improved MBLs were observed during the study. These results are in agreement with the results reported by Cappiello et al. and other authors who conducted studies over longer follow-up periods, thus indicating that PS reduced the resorption of the peri-implant crestal bone and increased the long-term success of implant therapy [1,34,40].

The present study showed positive results for using PS during implant therapy. Other studies have also demonstrated several potential disadvantages of PS, including the need for components that have similar designs and the need for enough space to develop an appropriate emergence profile [33]. This procedure shifts the concentration of stress away from the bone-implant interface, but these forces are subsequently increased within the abutment or the abutment screw [41]. Consequently, the dental implant used in this study was ideal for this approach due to its simple single-abutment system and internal conical connection between the implant and abutment interface, which eliminated any possible disadvantages associated with platform switching and maintained stability in platform matching during the study period.

## 5. Conclusions

PS implants appeared to be more effective for promoting slight gain in vertical and horizontal mean MBLs at three years when compared to PM implants. Despite the abutment connection used, the dental implant in the present study showed minimal levels of remodeling in the marginal bone, thus indicating a good prognosis for long-term treatment. Further studies with a longer follow-up and measurements of other parameters are needed to determine the effect of PS on surrounding bone tissue.

## Figures and Tables

**Table 1:** Demographic Information.

	Platform Matching	Platform Switching
**Age (years)**	53.95 ± 22.85	53.72 ± 21.85
**Sex**		
Male	15 (62.5%)	9 (60.87%)
Female	9 (37.5%)	14 (39.13%)
**Implant location**		
Anterior	4 (13.33%)	2 (6.67%)
Posterior	26 (86.67%)	28 (93.33%)
Maxilla	25 (83.33%)	13 (43.33%)
Mandible	5 (16.67%)	17 (56.67%)
**Implant diameter (mm)**		
3.6	5 (16.65%)	5 (16.65%)
4.0	14 (46.66%)	10 (33.33%)
4.5	10 (33.33%)	13 (43.33%)
5.0	1 (3.33%)	2 (6.66%)

**Table 2:** Marginal bone level for PS and PM implants at different time points (mean ± SD mm).

	Platform Switching		Platform Matching	
Time	Mean Vertical	Mean Horizontal	Mean Vertical	Mean Horizontal
**Before loading**	1.74 ± 1.32	1.32 ± 1.64	1.29 ± 0.90	1.35 ± 0.75
**Immediately after loading**	1.91 ± 1.15	1.36 ± 0.97	1.11 ± 0.72	1.29 ± 0.72
**1 month after loading**	1.74 ± 1.20	1.36 ± 0.92	1.10 ± 0.64	1.26 ± 0.70
**3 months after loading**	1.56 ± 1.17	1.17 ± 0.93	1.05 ± 0.82	1.23 ± 0.70
**6 months after loading**	1.10 ± 0.93	0.90 ± 0.59	0.96 ± 0.77	1.22 ± 0.70
**12 months after loading**	0.99 ± 0.95	1.16 ± 0.59	0.89 ± 0.58	1.00 ± 0.51
**36 months after loading**	0.78 ± 0.77	0.84 ± 0.45	0.98 ± 0.81	0.98 ± 0.68
